# Mechanisms driving vestibular lamina formation and opening in the mouse

**DOI:** 10.1111/joa.13771

**Published:** 2022-10-01

**Authors:** Tengyang Qiu, Abigail S. Tucker

**Affiliations:** ^1^ Centre for Craniofacial and Regenerative Biology, Faculty of Dentistry, Oral and Craniofacial Sciences King's College London London UK

**Keywords:** apoptosis, epithelial differentiation, oral mucosa, vestibule

## Abstract

The vestibular lamina (VL) forms as an epithelial outgrowth parallel to the dental lamina (DL) in the oral cavity. During late development, it opens to create a furrow that divides the dental tissue from the cheeks and lips and is known as the vestibule. Defects in this process lead to failure in the separation of the teeth from the lips and cheeks, including the presence of multiple frenula. In this paper, the development of the VL is followed in the mouse, from epithelial placode in the embryo to postnatal opening and vestibule formation. During early outgrowth, differential proliferation controls the curvature of the VL as it extends under the forming incisors. Apoptosis plays a role in thinning the deepest part of the lamina, while terminal differentiation of the epithelium, highlighted by the expression of loricrin and flattening of the nuclei, predates the division of the VL into two to create the vestibule. Development in the mouse is compared to the human VL, with respect to the relationship of the VL to the DL, VL morphology and mechanisms of opening. Overall, this paper provides insight into an understudied part of the oral anatomy, shedding light on how defects could form in this region.

## Graphical abstract

The mouse vestibular lamina (VL) extends as a thin sheet around the lower incisor region prenatally and opens postnatally to create a deep lower vestibule. Apoptosis thins the extending lamina while opening starts with the formation of small pockets during a process of terminal differentiation. Similarities and differences with human VL development are discussed.

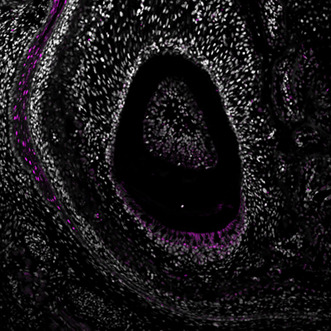

## 1 INTRODUCTION

The vestibular lamina (VL) is a transient mammalian structure that forms during embryonic development and creates the vestibule, the gap separating the teeth from the cheeks and lips. It has also been referred to as the lip furrow band and vestibuli oris (Bolk, 1921; Peterkova, 1985; Schour, 1929). In some human syndromes, such as EVC syndrome (OMIM 225500), the development of the VL is compromised, leading to defects in the vestibule, such as multiple frenuli causing labioginival adherences (Nakatomi et al., 2013; Sasalawad et al., 2013). Vestibule defects have also been noted in some ectodermal dysplasias, such as Weyers acrofacial dysostosis (also known as Weyers acrodental dysotosis) (OMIM 193530) (Roubicek & Spranger, 1984) and, most recently, VL abnormalities and dental anomalies were reported in a patient with cryptophthalmos resulting from a mutation in the FREM2 gene (OMIM 23570) (Kantaputra et al., 2022). Shallow vestibules were noted in 3.6% of healthy children in a study of 83 children, with a small number of frenulum abnormaliies observed (Kus‐Bartoszek et al., 2022). Such frenulum defects have been linked to later development of some types of periodontal disease (Placek et al., 1974).

In the embryo, the vestibular lamina forms in close relationship to the neighbouring dental lamina (DL). The DL goes on to form the tooth germs, and in some parts of the jaw the two laminas share a common placodal origin in both the mouse and human (Hovorakova et al., 2016; Peterkova, 1985; Qiu et al., 2020). The VL and DL are often thought of horse‐shoe structures with the VL running continuously around the jaw parallel to the DL, however in humans 3D reconstructions reveal that the VL is discontinuous with numerous connections to the DL (Hovorakova et al., 2005, 2007). Interestingly, in some positions along the jaw the VL forms tooth germ‐like structures, and in some mouse mutants tooth germs can develop from the VL, suggesting it has the potential to form teeth, similar to the DL (Popa et al., 2019; Wang et al., 2009). Some odontomas (tooth‐like tumours) are also associated with the VL in patients, providing further evidence that the VL has dormant tooth‐forming potential (Hovorakova et al., 2020).

Recently, the early development of the VL has been followed in human embryonic and foetal tissue, highlighting the relationship between the VL and DL and the process of opening to create the vestibule. In humans, the VL starts to open around 11 weeks by the creation of fissures due to differentiation of the epithelium and loss of epithelial integrity (Qiu et al., 2020). Apoptosis then plays a role in removing cells from the middle of the opening VL to create a large gap between the future teeth and cheeks (Qiu et al., 2020).

Morphology of the developing VL has been followed in a few mammals, such as the sheep, field vole and mouse, highlighting that there are clear species‐specific differences in the size and shape of the VL in different parts of the mouth. The morphology of the VL may therefore be shaped by later feeding requirements. The mouse and vole VL forms as a thin lamina and is prominent in the anterior mandible, while in humans and sheep the VL is multi‐layered and found associated with all teeth (Hovorakova et al., 2005, 2016; Pavlikova et al., 1999; Qiu et al., 2020; Witter et al., 2005). In the mouse, the early development of the VL has been followed using 3D reconstruction of the incisor region from Embryonic day (E)11.5 to E13.5, highlighting the close relationship of the tooth germ and neighbouring VL (Hovorakova et al., 2011). Later questions about how the VL extends and opens and the timing of such events have not been investigated. A lack of knowledge regarding VL development in the mouse has led to this structure being largely ignored in the description of mouse mutants and being missed out in recent schematics of the oral cavity (Ye et al., 2022).

Here the development of the murine VL has been followed from initiation in the embryo to vestibule formation during postnatal stages. The mechanisms that drive the extension and opening of the VL are described, highlighting the role of epithelial differentiation and distinct differences between the labial and lingual sides of the VL. Overall, this paper provides a systematic description of the formation of the vestibule, which will provide an essential source for further understanding of normal and abnormal development of this structure.

## 2 MATERIALS AND METHODS

### 2.1 Murine tissue collection

Mouse embryos were collected from wild‐type mice of CD1 strain, which were housed in the Biological Services Unit in New Hunts House at King's College London (KCL). Day 0.5 was considered midday on the day that a plug was found. Postnatal stages from P0 to P15 were collected from wild‐type mice (CD1 and C57Bl6 background). All animals were culled using schedule one culling methods as approved by the UK Home Office. For proliferation assays, pregnant dams were injected with Bromodeoxyuridine (BrdU) (30 mg/kg) 1 h before culling.

### 2.2 Tissue processing and histology

Dissected heads were fixed in 4% paraformaldehyde (PFA), dehydrated through an increasing ethanol concentration, before moving to xylene and embedding in paraffin wax. Postnatal tissues were decalcified in 0.5 M ethylenediaminetetraacetic acid (EDTA) before dehydration. Sections were cut in 8 μm serial sections using a Microtome Leica RM2245, and serially split onto slides. For histology trichrome staining was using: sirius red, Alcian blue and Hematoxylin. Stained slides were photographed using a Nikon Eclipse 80i light microscope attached with a Nikon Digital Sight DS‐Fi1 camera.

### 2.3 Explant culture

Wild‐type pregnant CD1 mice were collected at embryonic stage E12.5 (*n* > 3). The mandibles were dissected, and tongue removed before being chopped sagittally into 250 μm thick slices using a McIlwain tissue chopper (Alfaqeeh & Tucker, 2013). The slices with a clear DL/VL bud in the incisor region were selected. Explants were placed on permeable membranes (BD Falcon cell culture inserts, pore size 0.4 μm) over culture medium (DMEM‐Advanced Dulbecco Modified Eagle Medium F12, [Invitrogen]; 1% GlutaMAX [Invitrogen]; and 1% penicillin–streptomycin solution [10,000 units penicillin and 10 mg streptomycin/ml; Sigma‐ Aldrich]). Slices were photographed using a Leica dissecting microscope at day 0 of culture, and then cultured in a 5% CO_2_ at 37°C in an incubator for up to 3 days, with the culture medium changed every 1–2 days. Slices were photographed at regular intervals before fixation in 4% PFA. For whole mount immunofluorescence, explant slices were cultured with Bromodeoxyuridine (BrdU) at a concentration of 30uM for 2 h before fixation in 4% PFA for 40 min at RT.

### 2.4 Immunofluorescence/whole mount immunofluorescence

Wax‐embedded serial sections of the VL were de‐waxed, rehydrated and treated with citric acid (pH 6) antigen retrieval solution in a 92°C water bath followed by 10 min at room temperature. The slides were then incubated with rabbit Cleaved Caspase‐3 (1:200, Cell Signaling #9579), mouse anti‐E‐cadherin (1:400; Abcam, ab76055), rabbit anti‐Loricrin (1:400; Biolegend, PRB‐145P), rabbit anti‐Occludin (1:200, Abcam #ab31721) rabbit anti‐PCNA (1:400; Abcam #ab193965), and rat anti‐BrdU (1:500, Abcam # ab6326), overnight at 4°C. The sections were then incubated in Alexa Fluor™ donkey anti‐mouse 488 (1:500, Invitrogen #A21202), Alexa Fluor™ donkey anti‐rabbit 568 (1:500, Invitrogen #A10042) and Alexa Fluor™ donkey anti‐mouse 647 (1:500, Invitrogen #A31571) for 1 h at RT. Sections were mounted with Fluoroshield™ with DAPI (Sigma‐Aldrich #SLBV4269) and imaged with a Leica TCS SP5 confocal microscope. To test each antibody, controls were performed where the primary antibodies had been omitted in order to confirm specific staining. Each antibody was repeated at least three times, at different timepoints, using serial sections.

For whole mount immunofluorescence, fixed BrdU cultured explant slices were permeabilized with PBS Triton 0.5% (PBT) at RT for 1.5 h followed by trypsinization for 12 min on ice and incubation in blocking solution for 2 h. After blocking, slices were incubated in primary antibodies mouse anti‐E‐cadherin (1:400; Abcam, ab76055) and rat anti‐BrdU (1:500, Abcam # ab6326) overnight at 4°C. Next day, after washing in 0.5% PBT for 3 h at RT, the slices were incubated in the Alexafluor donkey anti‐rat 647 (1:500, Invitrogen, A21247), Alexa Fluor™ donkey anti‐mouse 488 (1:500, Invitrogen #A11001) and DAPI (1:1000, Sigma) overnight in the fridge. Finally, the slices were washed in 0.5% PBT for 3 h at RT, mounted in PBS and analysed using a Leica TCS SP5 confocal microscope.

### 2.5 Cell quantification and statistical analysis

Cells on the lingual and labial sides of the VL were quantified manually using the multiple‐point tools of Fiji/ImageJ. Results were plotted using GraphPad Prism software (GraphPad Prism V.8.0.2) and statistical analysis were performed using IBM SPSS Statistics software (IBM SPSS Statistics V.25.0). Statistical significance was calculated using paired *t*‐tests (comparing lingual and labial sides of the same VL). Significance was taken as *p* < 0.05 (*), *p* < 0.01 (**) or *p* < 0.001 (***).

## 3 RESULTS

### 3.1 Development of the mouse VL varies between the upper and lower jaw during development

**FIGURE 1:**
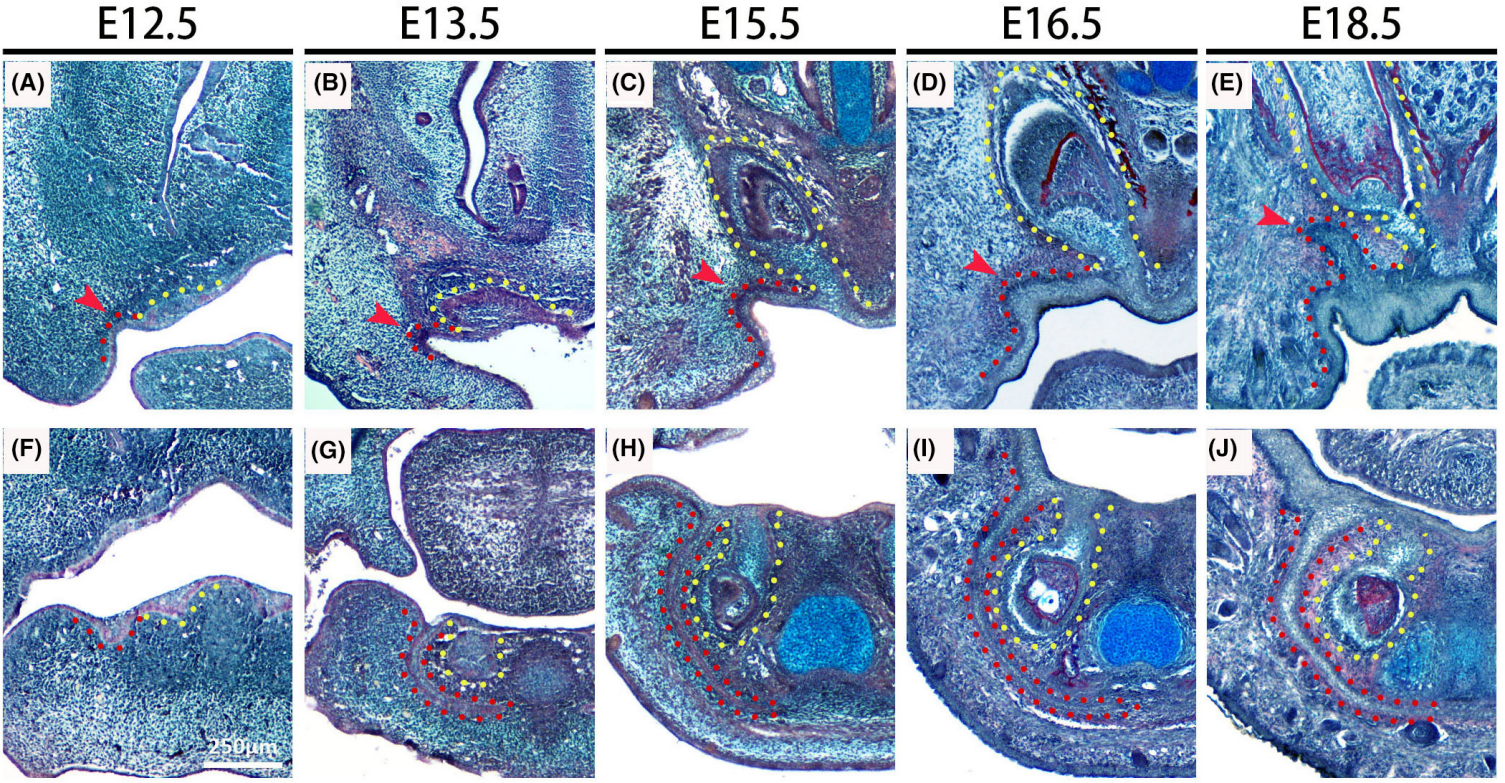
Embryonic development of the vestibular lamina (VL) in the murine incisor region. (A–E) Frontal sections of the upper jaw in wild‐type (WT) mouse embryos stained with trichrome. (F–J) Frontal sections of lower jaw in WT mouse embryos stained with trichrome. (A, F) E12.5, (B, G) E13.5, (C, H) E15.5, (D, I) E16.5, (E, J) E18.5. Dental lamina/incisors and VL are outlined by yellow and red dashed lines, respectively. Scale bar in (F) = 250 μm, same scale in all other figures

The VL is prominent in both the upper and lower jaw in humans (Hovorakova et al., 2005, 2007; Qiu et al., 2020). In the mouse, the VL is evident in the anterior of the mouth, but a clear structure was less obvious more posteriorly near the molars (Hovorakova et al., 2011; Peterkova, 1985). We therefore analysed the developing VL in the mouse focusing on the more anterior/incisor region from E (embryonic day) 12.5, the stage when the VL and DL are at the placodal thickening stage. In the anterior part of the upper jaw, only small areas of thickened oral epithelium were observed lateral to the forming DL/tooth primordia from E12.5 to E16.5, which were barely distinguishable from the oral epithelium (Figure 1A–D). At E18.5 the maxillary VL was more evident, forming an epithelial projection lateral to the forming incisors (Figure 1E). In contrast, in the mandible, the VL was much more pronounced at the same embryonic stages. At E12.5 two thickened laminas were evident, the VL positioned lateral to the DL (Figure 1F). By E13.5 the incisor had reached the bud stage, while the adjacent VL had extended down and around the forming tooth (Figure 1G). By E15.5 the VL had extended under Meckel's cartilage, with the two laminas almost touching in the midline by E16.5 (Figure 1H,I). The VL remained a solid structure at E18.5 (Figure 1J). In human embryos, the DL extends from the VL in both the upper and lower jaw (Figure S1; Qiu et al., 2020). In contrast, in the mouse the DL and VL both extended directly from the oral surface at all stages investigated.

### 3.2 Asymmetrical proliferation of the extending VL leads to bending of the outgrowth

**FIGURE 2:**
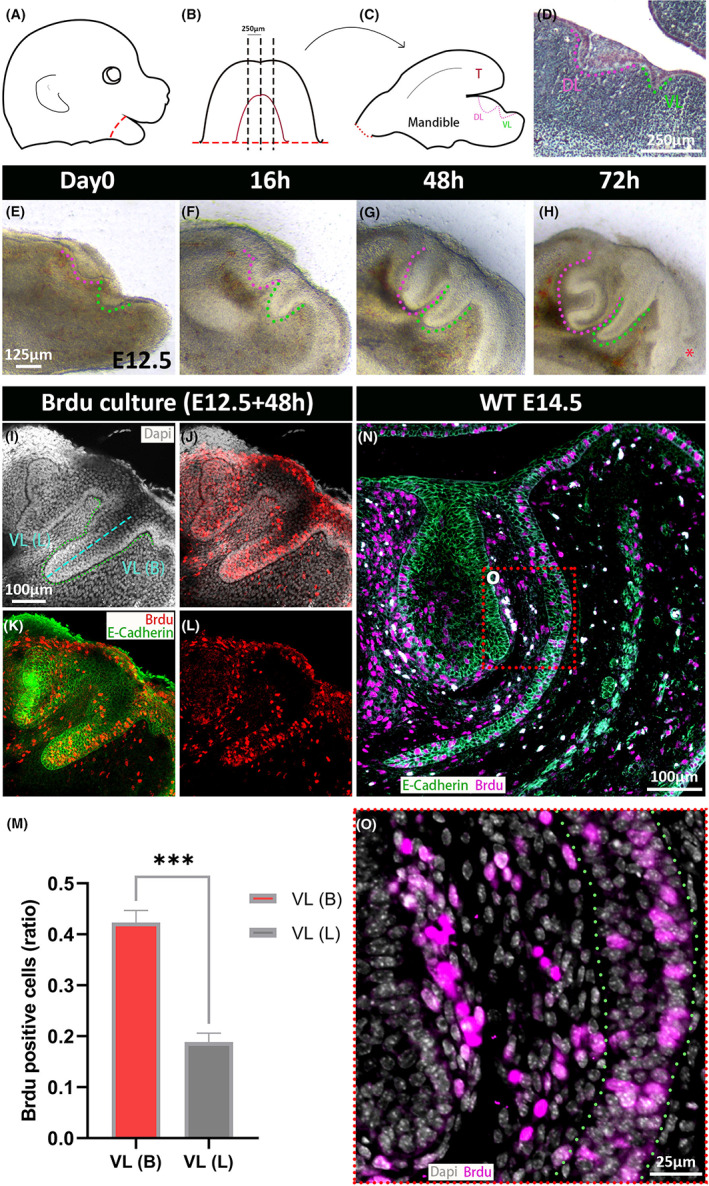
Differential proliferation shapes the developing VL. (A) Schematic of E12.5 mouse head. Red dots show the mandible dissection. (B) Diagram of dissected mandible. Dark lines indicate the chopping planes to create sagittal slices. (C) Medial slices present a tongue (T), and two protrusions, for the VL (green) and DL (magenta) respectively. (D) Histological sagittal section through a mouse embryonic head at E12.5. Magenta dashed line labels DL, and green dashed line outlines VL. (E–H) Developing sagittal slice of the lower jaw from E12.5. (E) Day 0. Two oral epithelial thickenings are prominent at E12.5. (F) Explant slices after 16 h in culture. (G, H) Slices after 48 and 72 h in culture. The VL extended into the mesenchyme and a clear tooth bud could be observed. Whiskers (red asterisks) also develop during the culture period. (I–L) Day 2 slice. Slices were cultured with BrdU for 2 h before fixing and processing for whole mount immunofluorescence. (I) DAPI (white nuclei). Green dashed line presents VL, light blue dashed line divides VL into lingual VL and buccal/labial VL. (J) DAPI (white) and BrdU (red). All BrdU expression is nuclear, confirming specificity. (K) BrdU (red) and E‐cadherin (green). (L) BrdU alone (red). (M) Statistical graph for BrdU‐positive cells (ratio) in the buccal VL compared to the lingual VL (*n* = 3, *p* < 0.001, error bars are SEM). (N, O) Frontal sections of lower jaw in wildtype mouse (E14.5). (N) BrdU (magenta) and E‐cadherin (green) outlines the incisor tooth germ and VL. (O) BrdU (magenta) and DAPI (white). Positive cells are evident on the outer edge of the VL. Scale bar in (D) = 250 μm. Scale bar in (E) = 125 μm, same scale in F–H. Scale bar in (I) = 100 μm, same scale in J–L, N. Scale bar in (0) = 25 μm. *** is the level of significance as *p* > 0.001. DL, dental lamina; VL, vestibular lamina; VL (B), Buccal VL; VL (L), lingual VL

In histological section, it was evident that the VL curves as it grows so that it extends under the tooth. This directional growth could be followed in slice culture. Here live sagittal slices of the jaw were generated at E12.5, when the VL was evident as a placode (Figure 2A–D). During the culture period, the incisor tooth germ (outlined in magenta) developed from a thickening to a late cap stage tooth, and the neighbouring VL (outlined in green) extended under the forming tooth (Figure 2E–H). To address what drives the VL extension, we explored the role of proliferation during VL development. We utilised BrdU as a marker for detecting the dividing cells during the S phase of the cell cycle. Explants were cultured with BrdU for 2 h at day 2 before fixation and processed for whole mount immunofluorescence (*N* > 3). The morphology of the VL and DL were highlighted by the epithelial cell adhesion marker E‐cadherin (Figure 2K). The developing slices had high levels of proliferation, as shown by BrdU‐positive cells (in red), with labelled cells in the epithelium and mesenchyme (Figure 2I–L). After counting it was evident that the labial/buccal side of the VL contained significantly more positive cells than the neighbouring lingual side, with differential proliferation of the epithelium potentially driving the shape of the VL (Figure 2M). To confirm this difference, BrdU‐positive cells were visualised in frontal sections at E14.5 (Figure 2N). As shown in culture, more positive cells (magenta) were observed on the buccal side of the VL compared to the lingual side near the tooth germ (Figure 2N,O). In section and in culture, the tip of the VL displayed high levels of proliferation throughout, driving the extension towards the midline (Figure 2L,N).

### 3.3 The VL opens postnatally to form the vestibule by P15

**FIGURE 3:**
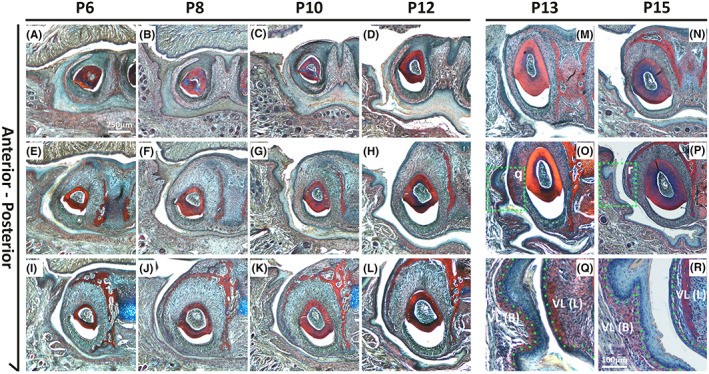
Opening of the VL postnatally to create the vestibule. (A–R) Frontal sections of lower jaw in wildtype (WT) mouse embryos stained with trichrome. (A, E, I) Postnatal day (P)6, (B, F, J) P8, (C, G, K) P10, (D, H, L) P12, (M, O, Q) P13, (N, P, R) P15. Anterior end of the VL around the incisors (A–D, M, N), Mid anterior region (E–H, O, P), posterior region (I–L). (Q, R) Higher power view of boxes in O, P to show differences in epithelial thickness on the buccal and lingual sides of the vestibule. Scale bar in (A) = 250 μm, same scale in B–P. Scale bar in (Q, R) = 100 μm. VL, vestibular lamina; VL (B), Buccal VL; VL (L), lingual VL

To follow the creation of the vestibule we analysed the opening process postnatally in the lower jaw. The VL opened in a wave from the posterior to the anterior. At postnatal day (P)6, the anterior VL was still solid, while more posteriorly it had already opened (Figure 3A,E,I). Interestingly the VL did not appear to open from the top in an unzipping mechanism, but holes appeared within the centre of the structure (Figure 3B–D,F,G). The deepest part of the VL, under the forming incisor teeth and Meckel's cartilage, was the last part of the VL to open. Splitting of the VL was complete anteriorly by P12‐P15, freeing the tooth‐forming region from the neighbouring cheeks (Figure 3D,H,L–N). Interestingly, the splitting of the VL into buccal and lingual sections was not symmetrical, with the buccal side consisting of several epithelial layers while the lingual side was composed of only a few cell layers (Figure 3O–R).

### 3.4 Postnatal opening of the VL does not appear to be driven by apoptosis

**FIGURE 4:**
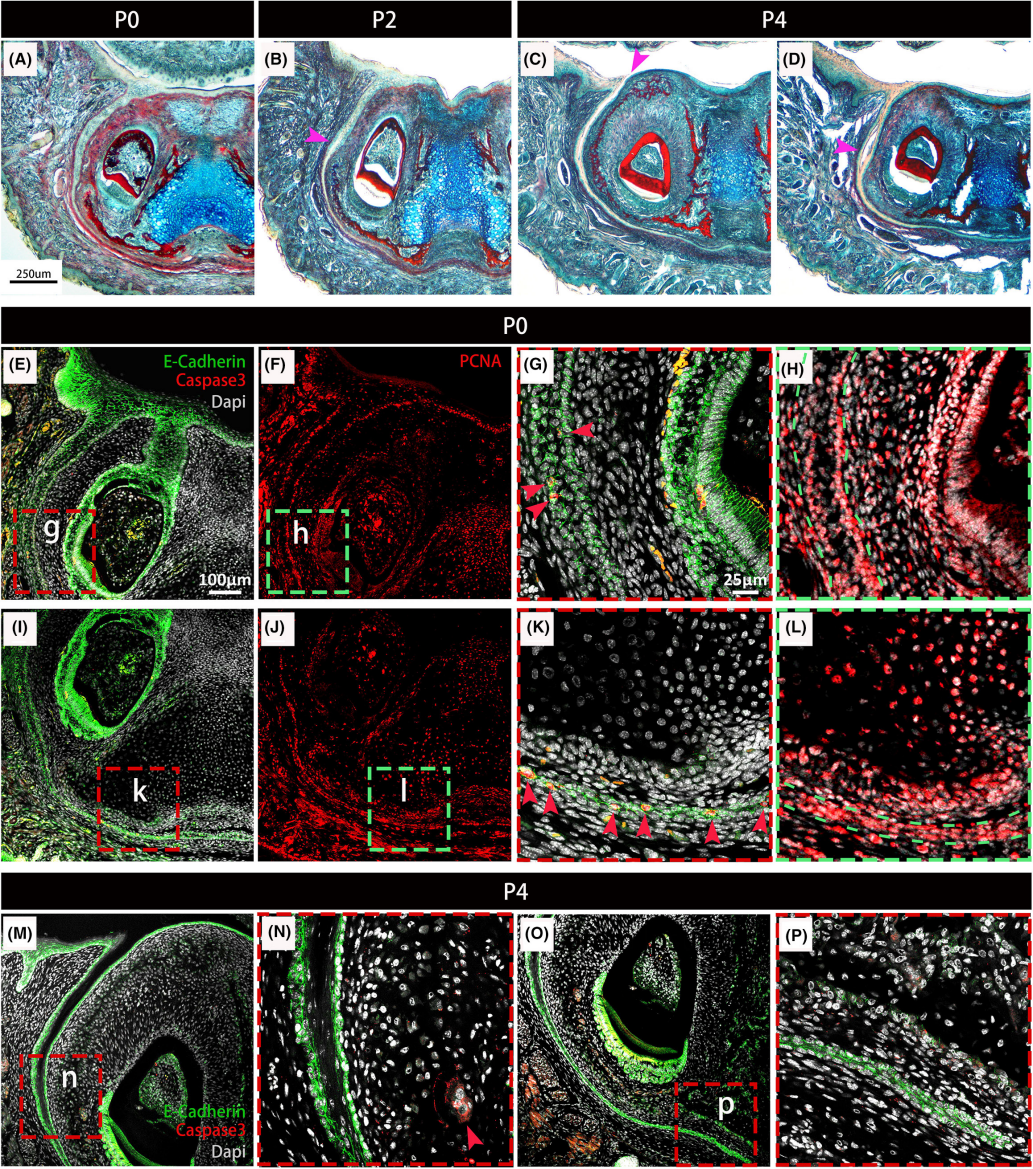
Apoptotic cells are not associated with cell clearance during VL opening. (A–D) Trichrome staining of the VL in the anterior lower jaw at P0, P2 and P4. Mouse frontal sections. C is more posterior than D, images taken from within the same mouse. (B) P2. Small holes form in the VL by P2, magenta arrowhead. (C, D) Fissures form in the VL by P4 (Magenta arrowheads), (C) forming from the oral surface, (D) forming as holes within the lamina. (E, G, I, K, M–P) Activated Caspase‐3 (red), E‐cadherin (green) and Dapi (grey) at P0 (E, G, I, K) and P4 (M–P) in the anterior VL in the murine mandible. (G, K) are higher magnifications of boxes (g, k) in (E, I). (F, H, J, L) PCNA (red) labels proliferating cells in the VL at P0. (H, L) are higher magnifications of boxes (h, l) in (F, J). Green dashes outline VL. (H) In the upper part of the VL PCNA cells are restricted to the basal cell layer L. (L) The lower VL is highly proliferative. A number of caspase3‐positive cells were found in the lower 2/3 of the VL (G), with high levels of positive cells at the end of the VL (K) at P0, at which point no obvious fissures were observed. At P4 no caspase3‐positive cells were observed throughout the whole lower VL, with only few in the mesenchyme on the lingual side of the VL (N). Red arrowheads indicate caspase3‐positive cells. Scale bar in (A) = 250 μm, same scale in B–D. Scale Bar in (E) = 100 μm, same scale in F, I, J, M, O. Scale Bar in (G ) = 25 μm, same scale in H, K, L, N, P. VL, vestibular lamina

**FIGURE 5:**
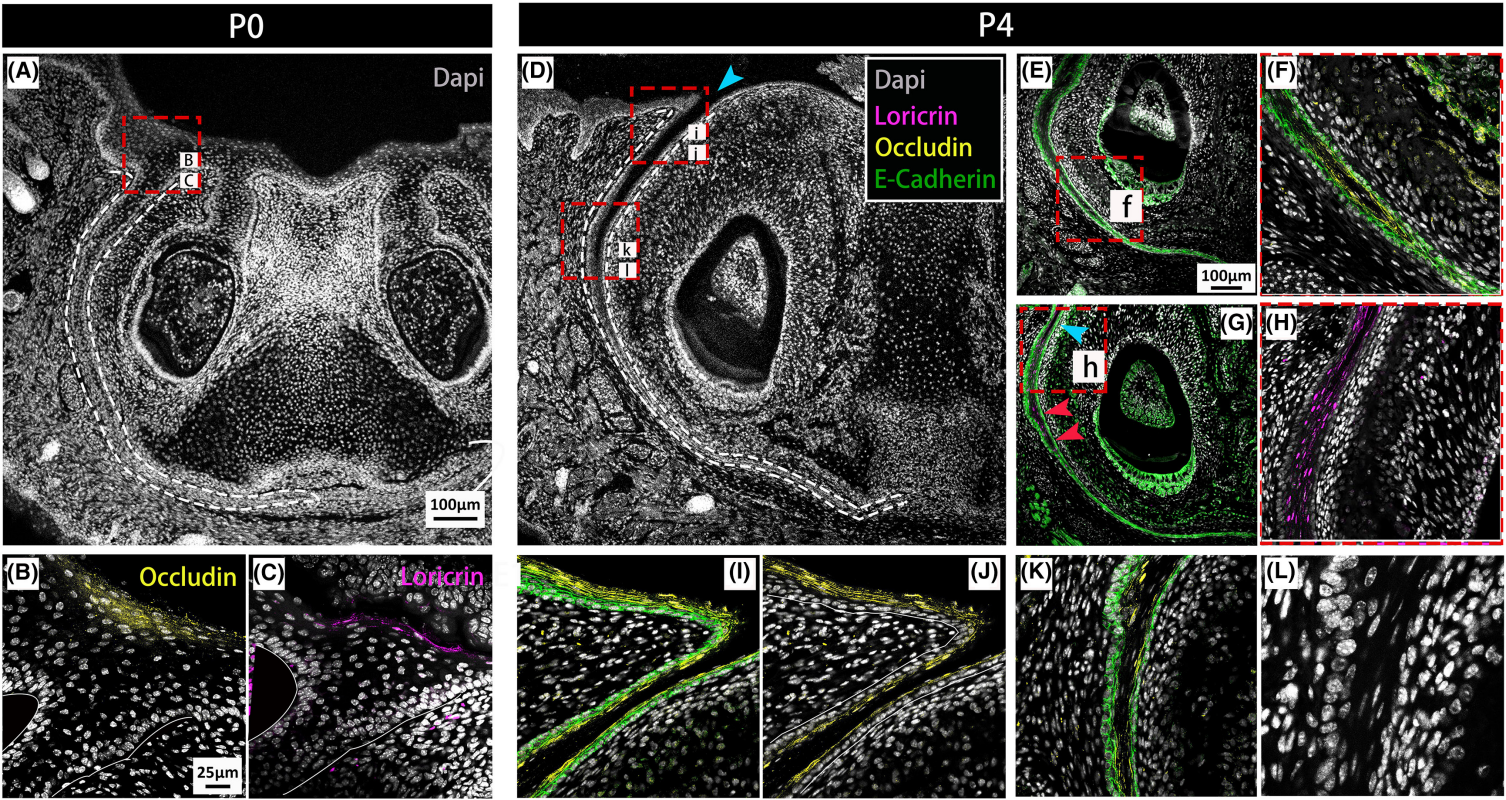
Opening of the VL postnatally is associated with terminal differentiation. (A–L) IF for DAPI (grey), Loricrin (magenta), E‐cadherin (green), occludin (yellow). (A–C) P0. (A) Anterior VL at P0. (B, C) Higher‐power magnification of the oral surface of the VL; occludin and loricrin are expressed in the granular layer and cornified layer, respectively. (D–L) P4. (D) Anterior VL at P4. (E, F, G, I, K) E‐cadherin labels the VL and incisor epithelium. Occludin‐positive cells surround a small opening within the middle of the VL (E, F, K) and line the opening VL at the top of the VL (I, J). (F) is a higher magnification of (f) in (E). (G, H) Loricrin‐positive cells were found in the suprabasal layer prior to opening. (H) is a higher magnification of (h) in (G); (L) highlights the deformation of the nuclei as they flatten in the centre of the VL; cyan arrowheads in (D) point to the main large furrow of the VL, and red arrowheads in (G) indicate small openings separate from the main fissure. Cyan arrows in (G) indicate the area with the main fissure. White dashed lines in (A, D) delineate the VL. Scale bar in (A) = 100 μm, same scale in D. Scales bar in (E) = 100 μm, same scale in F. Scale bar in (B) = 25 μm, same scale in C, F, H, K

To understand the opening process in more detail, we focused on P0 to P4, just as the VL is starting to change from a solid structure. At birth (P0), the VL remained solid (Figures 4A and 5A). The first sign of opening was the appearance of small holes in the VL at P2 (Figure 4B). By P4, small holes were observed within the deeper parts of the lamina, with the VL open additionally at the oral surface in more posterior sections (Figures 4C,D and 5D). Ecadherin (Ecad) was used to follow changes to the epithelium during this period. Already by P0 expression of Ecad was reduced in the centre (suprabasal layer) of the VL in the top two thirds of the structure, this becoming more pronounced by P4, where a central core of Ecad‐negative cells was observed (Figure 4M,N). The Ecad‐positive basal cells were PCNA positive, with reduced proliferation in the core of the VL (Figure 4F,H). The deep part of the VL had high levels of proliferating cells, highlighting that the VL is still growing at this stage (Figure 4J,L). Our previous study on VL opening in human embryos highlighted that apoptosis played a role in broadening the VL furrow but not in initial fissure formation, although localized cellular atrophy has been thought as the cause of the split in some research (Bolk, 1921; West, 1924). At early embryonic stages of development only scattered apoptotic bodies were observed in the vole VL (Witter et al., 2005). To clarify the mechanisms in the mouse, apoptosis was studied from P0 to P4 using activated caspase 3 as a marker. At P0, prior to opening, a few apoptotic cells were localised to the VL. In the main body of the VL, positive cells were scattered in the epithelium (Figure 4G), while at the tip of the VL many of the epithelial cells were positive (Figure 4K). In contrast, at P4 no apoptotic cells were observed in the VL, despite the presence of caspase‐positive cells in adjacent tissues (Figure 4M–P). Apoptosis was not associated with the formation of holes in the VL and did not appear to be a central driver for opening. As previously observed by histology, the tip of the VL does not open until P10 (Figure 3). The cells undergoing apoptosis in this region at P0 are, therefore, unlikely to have a role in opening of the VL, and instead may play a role in thinning of the epithelium in this region.

### 3.5 Terminal differentiation may trigger opening of the VL

The changes in Ecad expression prior to opening suggested changes in cell adhesion might be driving the opening process. In human embryos formation of fissures with the VL was linked to the onset of differentiation (Qiu et al., 2020). The identity of the central cells in the VL was therefore followed from P0 to P4 by immuno for occludin and loricrin. Occludin is an integral membrane protein identified at the tight junction and is used as a marker of the granular layer (Zihni et al., 2016). Loricrin is a terminal differentiation marker labelling the cornified layer of the skin (Ishitsuka & Roop, 2020; Koster & Roop, 2004). At P0, prior to any signs of opening, occludin and loricrin were restricted to the top of the VL, where the VL met the oral surface (Figure 5B,C). At P4, the open VL near the oral surface was lined by occludin‐positive cells, which formed a layer over the Ecad‐positive VL epithelium (Figure 5I,J). Further down the lamina, where the VL had yet to fully open, a similar arrangement was observed with Ecad outer cells flanking occludin positive inner cells around the forming holes (Figure 5E,F) and near to open regions (Figure 5K). The central (suprabasal) cells close to the oral surface were positive for the terminal marker loricrin (Figure 5G,H). During skin development, the skin undergoes terminal differentiation and cornification, a form of cell death (Eckhart et al., 2013). Cornification does not involve caspase 3, but other caspases such as caspase 14 are involved (Lippens et al., 2000). During terminal keratinocyte differentiation, the nuclei become flattened before nuclei degeneration (Eckhart et al., 2013). Interestingly, the nuclei in the centre of the VL had a distinctive flattened appearance, when compared to the rounded cells observed in the basal layers (Figure 5L). Loss of Ecadherin and the onset of occludin and loricrin, therefore, predated opening of the VL, with opening potentially linked to a process of cornification.

## 4 DISCUSSION

### 4.1 Species‐specific differences in VL morphology require distinct mechanisms for vestibule formation

The mouse VL was very different in morphology when compared to the human VL. The human VL was much wider, relatively, with the DL physically attached to it via epithelial bridges throughout the mouth (Qiu et al., 2020) (Figure S1). In contrast, the murine VL was very thin and only prominent in the anterior part of the lower jaw. The murine VL and DL, despite sharing an early common origin in the anterior region (Hovorakova et al., 2016), developed at a distance from each other, with separate connections to the oral cavity. Anteriorly, the murine VL extended much further into the oral cavity than the human VL, with the result that the murine vestibule around the lower incisors would be much deeper than in humans. In contrast, the murine upper incisors were associated with only a rudimentary VL and, therefore, would have a very shallow vestibule on the upper jaw. Such differences are likely to reflect differences in diet and manner of eating.

The different morphologies of the VL resulted in different mechanisms of opening. In human embryos, fissures developed in the wide VL as the epithelial cells underwent differentiation, with the central tissue removed by apoptosis (Qiu et al., 2020). In contrast, the suprabasal cells in the thin murine VL did not appear to be removed by apoptosis but underwent terminal differentiation and possible cornification.

**FIGURE 6:**
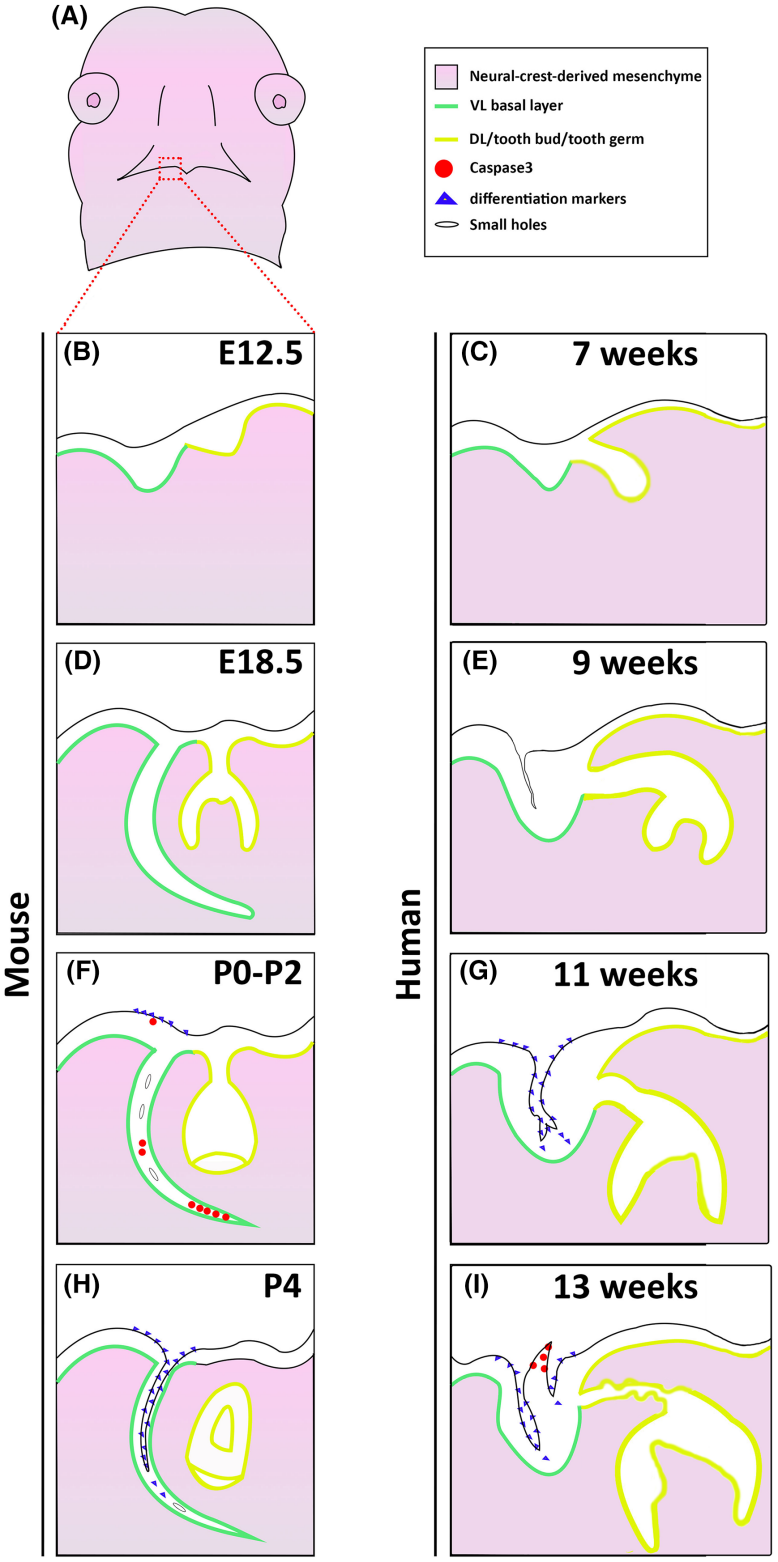
Comparison of vestibular lamina (VL) development and opening in human and mouse development. (A) Schematic of developing face highlighting area shown in B‐I. (B, D, F, H) Mouse incisor and VL. (C, E, G, I) Human incisor and VL. (B) E(embryonic day) 12.5. (D) E18.5, (F) P (postnatal day) 0‐2, (H) P4. (C) 7 weeks gestation, (E) 9 weeks, (G) 11 weeks, (J) 13 weeks

In human embryos, the fissures formed from the oral surface (Figure S1), while in the mouse, there was a combination of splitting of the VL near the oral surface and the formation of small holes along the length of the VL. This process of cavitation therefore shares some similarities with salivary gland lumen formation, where the ducts open by the coalescence of multiple small cavities (Tucker, 2007). The vestibule was completely open by postnatal day 13 to 15, which corresponds to the eruption of the dentition (first molar erupts at P15) and the move to a solid diet (Chlastakova et al., 2011). An incomplete vestibule, therefore, is not a problem for suckling in the first 2 weeks after birth. The differences and similarities in VL and DL formation in mouse and human are highlighted in Figure 6.

### 4.2 Lingual‐labial differences in the VL are evident from early stages of development

During growth of the murine VL proliferation was observed asymmetrically in the basal epithelial layer, so that more cells were positive on the labial/buccal side. This is predicted to cause a bend in the developing lamina, directing it under the forming tooth germ, so that the vestibule forms under the incisors. This difference in proliferation may also explain the observed asymmetrical split when the VL opened, with the labial side being much thicker. Due to these differences, the resulting mucosa of the oral cavity is therefore different on the side of the teeth and towards the cheeks. Such differences in thickness of the mucosa may result in differences in robustness, with the cheeks having more layers to counter the forces of chewing. Similar differences between the labial and lingual sides of the lamina were observed in human embryos (Qiu et al., 2020), with differences in keratin patterns on either side of the vestibule maintained in adult tissues (Verlach et al., 2017), suggesting this is a conserved mechanism.

### 4.3 Apoptosis and differentiation play distinct roles in VL development

The presence of activated caspase 3‐positive cells was not associated with opening of the murine VL at postnatal stages, however, large numbers of apoptotic cells were associated with the bottom third of the VL. These cells undergoing apoptosis were observed over a week before opening of this region and it is proposed that they have a role in thinning, rather than splitting of the VL. This may make later opening of the deep parts of the lamina easier. Opening was associated with loss of Ecadherin, reduced proliferation and upregulation of differentiation markers in the suprabasal layers of the VL. Occludin, as a functional component of tight junctions, marks the granular layer and turns off in the skin to allow shedding of the top layers (Zihni et al., 2016). Similarly, occludin turned off in the middle layers of the murine VL, as loricrin turned on, suggesting a change in cell adhesion. The loricrin expressing suprabasal cells had flattened nuclei, distinct from the rounder Ecad expressing basal cells. This change in cell morphology predated splitting of the VL. A similar change in cell morphology and upregulation of loricrin has been shown in the developing ear canal, another epithelial structure that goes from a solid lamina to an open tube (Fons et al., 2020). Terminal differentiation is therefore potentially a conserved mechanism to open epithelial tubes/laminae. The flattening of the nuclei may suggest that the canal opens due to cornification, with loss of adhesion and epithelial shedding, similar to the process observed in the skin. Cell death mechanisms, other than apoptosis, may therefore regulate this process (Eckhart et al., 2013). It would be interesting to identify whether markers associated with cornification, such as Caspase 14 were upregulated in these cells. Alternatively, the VL may open due to a loss of adhesion between the terminally differentiating cells without cell death. In the human VL, the upregulation of filigrin around the forming fissures suggests that downregulation of tight junctions might cause breaks to form within the epithelium. The murine VL showed upregulation of the terminal marker loricrin postnatally. Interestingly, in the adult oral cavity loricrin has been observed in the palatal but not the buccal epithelium (Ishitsuka & Roop, 2020), suggesting that after opening the loricrin population may not persist. Defects in the extension of the VL or opening of the deepest parts of the VL would be predicted to lead to the formation of a shallow vestibule. Likewise, incomplete separation along the VL could result in the formation of additional frenula, tethering the teeth to the cheeks and lips.

Overall, this paper has shed light on the development of a neglected structure that has an important role in creating the oral cavity. Here we provide an understanding of the timing of development and mechanisms of murine vestibule formation that can tbe used to understand defects associated with this region.

## ACKNOWLEDGEMENT

Thanks to Maria Hovorakova for discussions on VL development. Thanks to the Human Developmental Biology Resource for human embryonic and fetal tissue used in Supplementary Figure 1.

## AUTHOR CONTRIBUTIONS

Abigail S. Tucker conceived the idea. Tengyang Qiu performed the histology and immunohistochemistry and explant culture experiments. Abigail S. Tucker and Tengyang Qiu wrote the manuscript. All authors contributed to the article and approved the submitted version.

## FUNDING INFORMATION

This work was supported by the Grant Agency of the Czech Republic (18‐04859S to AT). TQ was funded by the China Scholarship Council as part of a PhD studentship at KCL.

## CONFLICT OF INTEREST

Abigail Tucker is a member of the Anatomical Society council, and sits on the editorial board of the Journal of Anatomy.

## Supporting information


Figure S1.

## Data Availability

The data that support the findings of this study are shown here and are available from the corresponding author upon request.
