# Supplementary figures and images for: Mechanisms driving vestibular lamina formation and opening in the mouse
Source: J Anat. 2022 Oct 1;242(2):224–34. doi: 10.1111/joa.13771 (PMC9877475; doi:10.1111/joa.13771)

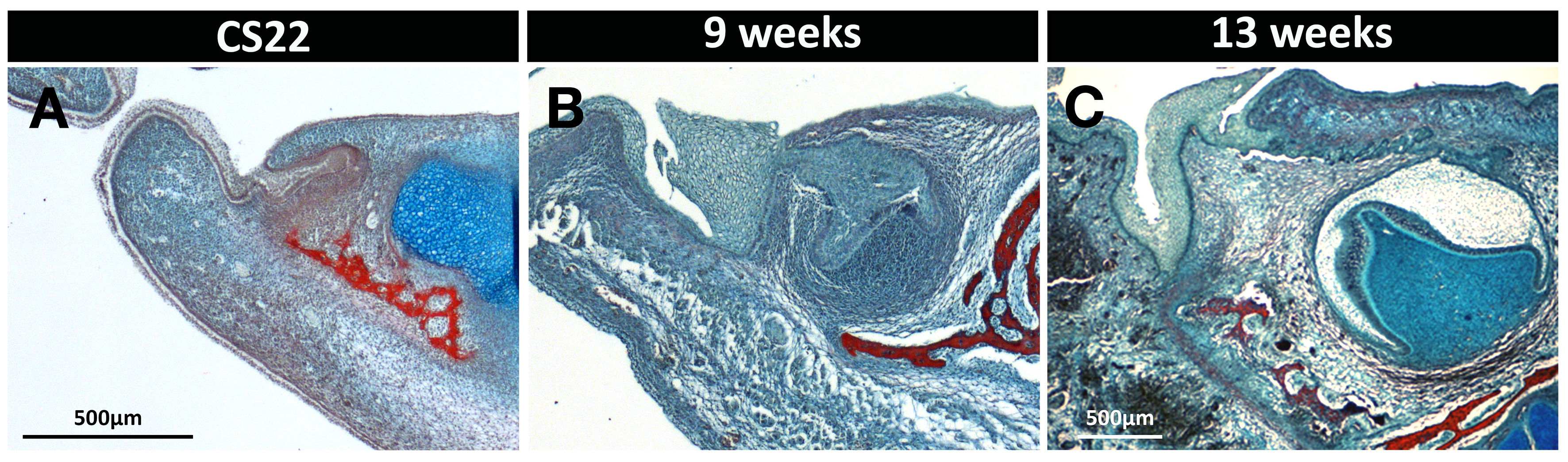

Supplement: Supplementary file 1 — Figure S1. [file JOA-242-224-s001.tif]
